# The Injection of Oil of Turpentine in Old Sinews

**Published:** 1887-01

**Authors:** 


The Injection of Oil of Turpentine into Old Sinuses.—Cecchini (*Anuali universali di medicina; Abeille méd.*) reports a number of cases in which he succeeded in causing the closure of old sinuous tracts by injecting into them a few drops of oil of turpentine with a common hypodermic syringe. The best results, he says, are obtained when the drug is used pure, but it may be mixed with some bland oil, or even with a solution of morphine, as the pain is something considerable. By this simple treatment, the author has cured five anal fistulæ and six sinuses connected with carious bone. The turpentine is thought to exert a stimulating action on the walls of the sinus, whereby healthy granulation is promoted.—*N. Y. Med. Journal.*

